# Identifying vital sign trajectories to predict 28-day mortality of critically ill elderly patients with acute respiratory distress syndrome

**DOI:** 10.1186/s12931-023-02643-8

**Published:** 2024-01-04

**Authors:** Mingzhuo Li, Fen Liu, Yang Yang, Jiahui Lao, Chaonan Yin, Yafei Wu, Zhongshang Yuan, Yongyue Wei, Fang Tang

**Affiliations:** 1 Department of Critical Care Medicine, Shandong Medicine and Health Key Laboratory of Emergency Medicine, Shandong Institute of Anesthesia and Respiratory Critical Medicine, The First Affiliated Hospital of Shandong First Medical University & Shandong Provincial Qianfoshan Hospital, Jingshi Road 16766, Jinan, China; 2 Center for Big Data Research in Health and Medicine, The First Affiliated Hospital of Shandong First Medical University & Shandong Provincial Qianfoshan Hospital, Jinan, China; 3 Shandong Data Open Innovative Application Laboratory, Jinan, China; 4 Institute of Pathogen Biology, Chinese Academy of Medical Sciences & Peking Union Medical College, Beijing, China; 5 Department of Biostatistics, School of Public Health, Cheeloo College of Medicine, Shandong University, Jinan, China; 6 Institute for Medical Dataology, Cheeloo College of Medicine, Shandong University, Jinan, China; 7 Department of Biostatistics, Center for Global Health, School of Public Health, Nanjing Medical University, Nanjing, China; 8 Shandong Provincial Qianfoshan Hospital, Cheeloo College of Medicine, Shandong University, Jinan, China

**Keywords:** Trajectory, Respiratory rate-oxygenation, 28-day mortality, Critical care, Precision medicine

## Abstract

**Background:**

The mortality rate of acute respiratory distress syndrome (ARDS) increases with age (≥ 65 years old) in critically ill patients, and it is necessary to prevent mortality in elderly patients with ARDS in the intensive care unit (ICU). Among the potential risk factors, dynamic subphenotypes of respiratory rate (RR), heart rate (HR), and respiratory rate-oxygenation (ROX) and their associations with 28-day mortality have not been clearly explored.

**Methods:**

Based on the eICU Collaborative Research Database (eICU-CRD), this study used a group-based trajectory model to identify longitudinal subphenotypes of RR, HR, and ROX during the first 72 h of ICU stays. A logistic model was used to evaluate the associations of trajectories with 28-day mortality considering the group with the lowest rate of mortality as a reference. Restricted cubic spline was used to quantify linear and nonlinear effects of static RR-related factors during the first 72 h of ICU stays on 28-day mortality. Receiver operating characteristic (ROC) curves were used to assess the prediction models with the Delong test.

**Results:**

A total of 938 critically ill elderly patients with ARDS were involved with five and 5 trajectories of RR and HR, respectively. A total of 204 patients fit 4 ROX trajectories. In the subphenotypes of RR, when compared with group 4, the odds ratios (ORs) and 95% confidence intervals (CIs) of group 3 were 2.74 (1.48–5.07) (*P* = 0.001). Regarding the HR subphenotypes, in comparison to group 1, the ORs and 95% CIs were 2.20 (1.19–4.08) (*P* = 0.012) for group 2, 2.70 (1.40–5.23) (*P* = 0.003) for group 3, 2.16 (1.04–4.49) (*P* = 0.040) for group 5. Low last ROX had a higher mortality risk (*P* linear = 0.023, *P* nonlinear = 0.010). Trajectories of RR and HR improved the predictive ability for 28-day mortality (AUC increased by 2.5%, *P* = 0.020).

**Conclusions:**

For RR and HR, longitudinal subphenotypes are risk factors for 28-day mortality and have additional predictive enrichment, whereas the last ROX during the first 72 h of ICU stays is associated with 28-day mortality. These findings indicate that maintaining the health dynamic subphenotypes of RR and HR in the ICU and elevating static ROX after initial critical care may have potentially beneficial effects on prognosis in critically ill elderly patients with ARDS.

**Supplementary Information:**

The online version contains supplementary material available at 10.1186/s12931-023-02643-8.

## Introduction

Acute respiratory distress syndrome (ARDS) is a common cause of respiratory system failure in critically ill patients [1], leading to noncardiogenic pulmonary edema [1] and increased permeability of the alveolar-capillary membrane. A previous study revealed that 10% of patients in all intensive care units (ICUs) met the ARDS criteria [2]; moreover, the mortality rate of ARDS increased with age (≥ 65 years old) [3, 4], reaching as high as 43% in ICU patients ≥ 67 years [4]. It is necessary to prevent mortality in elderly patients with ARDS in the ICU.

The respiratory rate (RR) is a crucial ventilatory parameter, despite being infrequently incorporated into ventilatory protocols in preclinical and clinical studies [5–7]. Protective mechanical ventilation strategies employing low tidal volume often result in an elevated RR to maintain adequate alveolar ventilation [8, 9]. However, there is a lack of conclusive data regarding the safety of high respiratory rates in preventing ventilator-induced lung injury [10]. Vieillard-Baron et al. [9] observed that a high RR in ARDS patients did not improve carbon dioxide (CO_2_) clearance. Instead, it led to dynamic hyperinflation and impaired right ventricular ejection. In addition, recent studies have affirmed a positive association between RR and mortality [8, 11]. Moreover, other indices associated with RR also have important implicaitons. For instance, heart rate (HR), which increases with RR, has been positively linked with mortality in elderly patients [12]. Navarrete-Navarro et al. [13] demonstrated that the ICU mortality in trauma patients with ARDS was related to oxygen partial pressure (PaO_2_)/fractional oxygen (FiO_2_) on the third day. Nonetheless, there has been widespread adoption of the respiratory rate-oxygenation (ROX), calculated as the ratio of oxygen saturation (SpO_2_)/FiO_2_ to RR. It is a more comprehensive indicator for predicting disease risk and prognosis [14, 15]. The higher ROX index at 24 h after initiating ventilator support was associated with lower mortality in patients with ARDS [16].

In summary, RR, HR, and ROX are readily monitored items in the ICU that can indicate clinical deterioration [11, 12, 16–18]. These parameters may serve as prognostic indicators for elderly patients with ARDS. However, there remain unresolved challenges. First, the implications of their variations have not been well studied. For ARDS patients in the ICU, their disease status could rapidly evolve within several minutes. This could be potentially signaled by simultaneous alterations in RR, HR, and ROX as a warning. Evidence supports that subclassifications of disease trajectories based on clinical biomarkers could identify typical dynamic subphenotypes in critically ill patients [10, 19–21, 30–32]. Second, linear and nonlinear effects of baseline and final readings of these indices on mortality were not investigated. The immediate responses of RR, HR, and ROX to critical care may have particular implications in mortality.

The objectives of this study are outlined as follows: (1) to identify dynamic trajectories of RR, HR, and ROX in elderly patients with ARDS during the initial 72 h of ICU admission using data from the eICU Collaborative Research Database (eICU-CRD); (2) to evaluate the relationship between these vital sign trajectories and 28-day mortality; (3) to examine both linear and nonlinear relationships of static levels of baseline and final measurements of RR, HR, and ROX during the first 72 h of ICU stays with 28-day mortality; and (4) to use a receiver operating characteristic (ROC) curve and area under the curve (AUC) to assess prediction models that incorporate significant trajectory factors and static RR-related factors.

## Materials and methods

### Study population and data sources

The study population was collected from the eICU-CRD 2.0 (year 2014–2015) [19] at PhysioNet [20, 21]. Patients with ICU stays > 72 h were first included, and the exclusion criteria were as follows: (1) no vital signs in the first 3 days of the ICU stay; (2) age < 65 or age ≥ 89; (3) gender unknown; (4) repeated measurements of RR and HR < 4 times during the first 72 h in the ICU; (5) no ARDS recorded; and (6) diagnosed with congestive heart failure. For screening ICU patients to fit trajectories of ROX, patients with repeated measurements of ROX < 4 times during the first 72 h in the ICU were excluded.

The international Classification of Diseases, Ninth Revision, Clinical Modification (ICD-9-CM) and the International Classification of Diseases, Tenth Revision, Clinical Modification (ICD-10-CM) were used to help define ARDS [22, 23]. We selected patients with ARDS using ICD-9-CM codes 518.51, 518.52, 518.53, and 518.82 [24, 25], ICD-10-CM code J80, and disease names including “ARDS” or “acute respiratory distress”. We included patients with ARDS diagnosis in the time range of 2 days before admission to the ICU and 1 day after admission. After screening target patients, 938 critically ill elderly patients with ARDS were collected from the eICU-CRD database.

### Demographic and clinical features

Demographic and clinical features were derived from 6 components: demographic information, severity of illness, support within the first 24 h, laboratory information, Charlson comorbidity, and vital signs. The detailed demographic and clinical features were as follows: (1) demographic information included age, sex, ethnicity, and first ICU location, and other or unknown conditions of ethnicity were regarded as a separate classification; (2) severity of illness included Sequential Organ Failure Assessment (SOFA) score, Acute Physiology Score III (APS-III), and Glasgow Coma Scale (GCS) score; (3) support during the first 24 h of ICU stays involved vasopressin, ventilation, and dialysis; and (4) laboratory information included baseline levels of laboratory indicators that were collected from the initial observations during the first 72 h in the ICU or were supplemented by the values closest to the time of the ICU stays before ICU admission. Detailed laboratory indicators were hemoglobin, platelets, white blood cells (WBCs), international normalized ratio (INR), partial thromboplastin time (PTT), blood urea nitrogen (BUN), creatinine, sodium, potassium, calcium, chloride, glucose, and bicarbonate; (5) Charlson comorbidity included myocardial infarct, congestive heart failure, peripheral vascular disease, cerebrovascular disease (CVD), dementia, chronic pulmonary disease (COPD), rheumatic disease, peptic ulcer disease, mild liver disease, severe liver disease, diabetes, paraplegia, renal disease, malignant cancer, metastatic solid tumor, and aids; and (6) vital signs included RR, HR, and ROX. Vital signs from hour 1 to hour 72 were split into one-hour blocks of time. If there were multiple measurements within one block, the peak values of RR and HR were used. For ROX, we first defined the minimum SpO_2_, maximum FiO_2_, and maximum RR in each one-hour block, and the minimum ROX was then calculated as the ratio of SpO_2_/FiO_2_ to RR [14]. The first observed vital signs were considered as baseline levels, whereas the vital signs finally observed during the first 72 h in the ICU were considered as post-treatment levels.

### Outcome

In the cohort, the follow-up started after 3 days in the ICU and lasted until death, loss to follow-up, or survival. Outcomes were defined as 28-day mortality, and 28 days were calculated from the fourth day of ICU admission.

### Statistical analysis

The characteristics of the eICU-CRD cohort were summarized using the mean ± standard deviation (SD), median (lower quartile-upper quartile), or number (proportion, %). Continuous variables of the trajectory groups were compared using t test or ANOVA for normally distributed data, and were compared using Wilcoxon rank sum test or Kruskal Wallis test for nonnormal data. Categorical variables of trajectory groups were compared using the chi-square test.

Latent mixture modeling (PROC TRAJ) was utilized to perform a group-based trajectory model (GBTM) [26] to identify the trajectories of RR, HR, and ROX. On the basis of prior studies [11, 27, 28], 2 to 5 trajectories were fitted using a linear and quadratic trajectory function based on a censored normal model [29] with age and sex adjustments. The average posterior probability of individuals belonging to each specific trajectory group was calculated, and the percentage of members in each trajectory group was presented. We selected the optimal model with the smallest absolute value of the Bayesian information criterion (BIC). The average posterior probability of each trajectory group was needed to be no less than 70%, and the percentage of members in each trajectory group was expected to be no less than 5%. Upon analyzing the trajectories of RR and HR, we noted that the patterns and numbers of trajectories of RR and HR were quite similar. Consequently, we calculated the Kappa statistic of the groups of RR and HR to evaluate the consistency of trajectory classification to avoid redundant analyses.

A logistic model was used to evaluate the relationships between trajectories and 28-day mortality in the univariate model. Statistically significant variables in the comparison of survivors and nonsurvivors were further adjusted in the multivariate model, and covariates with missing rates > 10% were not included. The trajectory group with lowest rate of 28-day mortality for each indicator was considered as a reference object in the logistic model.

Restricted cubic spline with 4 knots (5%, 35%, 65%, and 95%) was fitted to calculate the linear and nonlinear associations of baseline and last RR, HR, and ROX in the first 72 h of ICU stays with 28-day mortality, and statistically significant variables in the comparison of survivors and nonsurvivors were adjusted.

In the full population, ROC and AUC were used to assess the predictive ability of the general logistic model (model 1 with adjustment for statistically significant variables in the comparison of survivors and nonsurvivors) and trajectory adjusted logistic model (model 2 with adjustment for variables in model 1 plus significant trajectory risk factors). In the population with at least 4 records of ROX, model 1, model 2, and another model 3 (with adjustment for variables in model 1 plus significant static RR related risk factors) were established. The Delong test was used to compare model 1 and model 2, model 1 and model 3.

All analyses were performed with SAS 9.4 and R 4.0.2. A two-sided *P* value less than 0.05 was considered statistically significant.

## Results

A total of 938 inpatients and 67,536 observations were involved in further analyses. Additional file 1: Fig. S1 displays the flow diagram illustrating the selection process of the population of the eICU-CRD database. The demographic information and clinical features grouped by mortality outcome are presented in Table 1. There were 748 survivors and 190 nonsurvivors (28-day mortality = 20.26%) with a median age of 74.00 [69.00, 80.00]. Compared with survivors, nonsurvivors exhibited higher rates of male sex (*P* = 0.014), vasopressin (*P* < 0.001), and ventilation (*P* = 0.002) and had a higher age (*P* < 0.001) and higher levels of SOFA (*P* < 0.001), APS-III (*P* < 0.001), GCS (*P* = 0.003), WBCs (*P* < 0.001), INR (*P* = 0.006), BUN (*P* < 0.001), creatinine (*P* = 0.002), and RR (*P* = 0.015). Among these significantly different variables, age, sex, SOFA, APS-III, vasopressin, ventilation, WBCs, BUN, creatinine, and RR which had low missing rates (≤ 10%) were adjusted in the further logistic regression models, RCS models, and models predicting 28-day mortality. Five RR trajectories, 5 HR trajectories, and 4 ROX trajectories were identified. The model fitting process of the trajectories is shown in Additional file 1: Table S1. The Kappa value equals to 0.102 (*P* < 0.001), which shows a poor consistency of trajectory classification of RR and HR.

**Table 1:** Description of baseline variables grouped by 28-day mortality for ICU patients

Variables	Survivor (n = 748)	Nonsurvivor (n = 190)	*P*
Demographic information			
Age, year	74.00 [69.00, 79.00]	78.00 [72.00, 83.00]	< 0.001
Sex, n (%)			0.014
Male	360 (48.13)	111 (58.42)	
Female	388 (51.87)	79 (41.58)	
Ethnicity, n (%)			0.264
Caucasian	588 (78.61)	163 (85.79)	
African American	66 (8.82)	12 (6.32)	
Asian	8 (1.07)	2 (1.05)	
Hispanic	37 (4.95)	8 (4.21)	
Native American	6 (0.80)	1 (0.53)	
Other/Unknown	43 (5.75)	4 (2.11)	
First ICU location, n (%)			0.131
MICU	98 (13.10)	25 (13.16)	
CCU-CTICU	59 (7.89)	14 (7.37)	
NICU	36 (4.81)	10 (5.56)	
Med-Surg ICU	421 (56.28)	112 (58.95)	
Cardiac ICU	57 (7.62)	11 (5.79)	
CTICU	21 (2.81)	1 (0.53)	
SICU	48 (6.42)	10 (5.26)	
CSICU	8 (1.07)	7 (3.68)	
Severity of illness			
SOFA	6.03 (2.63)	7.30 (2.74)	< 0.001
APS-III	50.00 [35.00, 68.00]	63.50 [46.25, 83.75]	< 0.001
GCS	13.00 [10.00, 15.00]	11.00 [10.00, 15.00]	0.003
Support within the first 24 h			
Vasopressin, n (%)			< 0.001
No	634 (84.76)	139 (73.16)	
Yes	114 (15.24)	51 (26.84)	
Ventilation, n (%)			0.002
No	253 (33.82)	51 (21.58)	
Yes	495 (66.18)	149 (78.42)	
Dialysis, n (%)			> 0.999
No	725 (96.93)	184 (96.84)	
Yes	23 (3.07)	6 (3.16)	
Laboratory information			
Hemoglobin, g/dL	10.68 (2.22)	10.38 (2.05)	0.081
Platelets, 10^9^/L	200.50 [149.25, 262.25]	193.50 [144.25, 273.00]	0.735
WBCs, 10^9^/L	11.40 [7.93, 15.70]	13.21 [9.40, 19.23]	< 0.001
INR, %	1.20 [1.10, 1.50]	1.30 [1.10, 1.80]	0.006
PTT, s	32.50 [28.00, 40.00]	34.60 [28.90, 43.08]	0.088
BUN, mg/dL	25.00 [17.00, 38.50]	31.00 [21.25, 55.00]	< 0.001
Creatinine, mg/dL	1.08 [0.76, 1.64]	1.30 [0.80, 2.21]	0.002
Sodium, mmol/L	138.73 (5.92)	139.12 (6.78)	0.472
Potassium, mmol/L	4.16 (0.75)	4.18 (0.72)	0.692
Calcium, mg/dL	8.25 (0.78)	8.29 (1.23)	0.722
Chloride, mmol/L	103.59 (7.28)	103.82 (7.97)	0.728
Glucose, mg/dL	141.00 [111.00, 180.00]	140.00 [111.25, 182.50]	0.934
Bicarbonate, mmol/L	25.45 (6.16)	24.50 (6.36)	0.073
Charlson comorbidity			
Myocardial infarct, n (%)			0.141
No	730 (97.59)	181 (95.26)	
Yes	18 (2.41)	9 (4.74)	
Peripheral vascular disease, n (%)			0.322
No	740 (98.93)	190 (100.00)	
Yes	8 (1.07)	0 (0.00)	
Cerebrovascular disease, n (%)			0.430
No	712 (95.19)	184 (96.84)	
Yes	36 (4.81)	6 (3.16)	
Dementia, n (%)			0.059
No	725 (96.93)	178 (93.68)	
Yes	23 (3.07)	12 (6.32)	
Chronic pulmonary disease, n (%)			0.870
No	597 (79.81)	150 (78.95)	
Yes	151 (20.19)	40 (21.05)	
Rheumatic disease, n (%)			> 0.999
No	745 (99.60)	189 (99.47)	
Yes	3 (0.40)	1 (0.53)	
Peptic ulcer disease, n (%)			0.772
No	744 (99.47)	188 (98.95)	
Yes	4 (0.53)	2 (1.05)	
Mild liver disease, n (%)			0.075
No	735 (98.26)	182 (95.79)	
Yes	13 (1.74)	8 (4.21)	
Severe liver disease, n (%)			> 0.999
No	745 (99.60)	190 (100.00)	
Yes	3 (0.40)	0 (0.00)	
Diabetes, n (%)			0.766
No	657 (87.83)	169 (88.95)	
Yes	91 (12.17)	21 (11.05)	
Paraplegia, n (%)			> 0.999
No	747 (99.87)	190 (100.00)	
Yes	1 (0.13)	0 (0.00)	
Renal disease, n (%)			0.276
No	669 (89.44)	164 (86.32)	
Yes	79 (10.56)	26 (13.68)	
Malignant cancer, n (%)			> 0.999
No	689 (92.11)	175 (92.11)	
Yes	59 (7.89)	15 (7.89)	
Metastatic solid tumor, n (%)			> 0.999
No	742 (99.20)	189 (99.47)	
Yes	6 (0.80)	1 (0.53)	
Aids, n (%)			0.867
No	747 (99.87)	189 (99.47)	
Yes	1 (0.13)	1 (0.53)	
Vital signs			
RR, /min	24.55 (8.00)	26.16 (8.19)	0.015
HR, /min	95.71 (22.55)	97.64 (21.17)	0.268
ROX	6.10 [3.92, 9.74]	5.65 [4.35, 8.38]	0.265

Data are presented using mean (standard deviation (SD)), median (lower quartile-upper quartile), or number (proportion, %)

*ICU* intensive care unit, *SOFA* Sequential Organ Failure Assessment, *APS-III* Acute Physiology Score III, *GCS* Glasgow Coma Scale, *WBCs* white blood cells, *INR* international normalized ratio, *PTT* partial thromboplastin time, *BUN* blood urea nitrogen, *RR* respiratory rate, *HR* heart rate, *ROX* respiratory rate-oxygenation

Additional file 1: Tables S2, S3, and S4 present the demographic information and clinical features of the different trajectory groups. In terms of respiratory rate trajectories, outstanding observations include the following: group 1 showed higher incidence of cerebrovascular disease, group 2 displayed elevated bicarbonate level, group 3 had increased rate of myocardial infarction, group 4 exhibited older age, higher GCS score, and elevated INR level, and group 5 had increased RR and HR. Notably, groups 1 and 2 were marked by higher ROX, groups 1 and 3 received higher rate of ventilation, and groups 3 and 5 experienced a higher mortality rate, whereas group 4 had a lower mortality rate. For HR trajectories, highlighted observations include the following: group 1 demonstrated a higher rate of ventilation, group 3 had elevated WBCs, group 5 exhibited higher APS-III, and both groups 1 and 5 displayed elevated creatinine level. Groups 4 and 5 were characterized by higher RR and HR, groups 1 and 3 received a higher rate of ventilation, and group 1 had a lower mortality rate. Regarding ROX trajectories, the main differences were as follows: group 1 displayed a higher SOFA score, an increased rate of cerebrovascular disease, and a higher prevalence of malignant cancer. This group also showed higher RR and HR. Group 4 comprised a higher proportion of females, and group 5 exhibited higher levels of potassium and ROX.

**Fig. 1:**
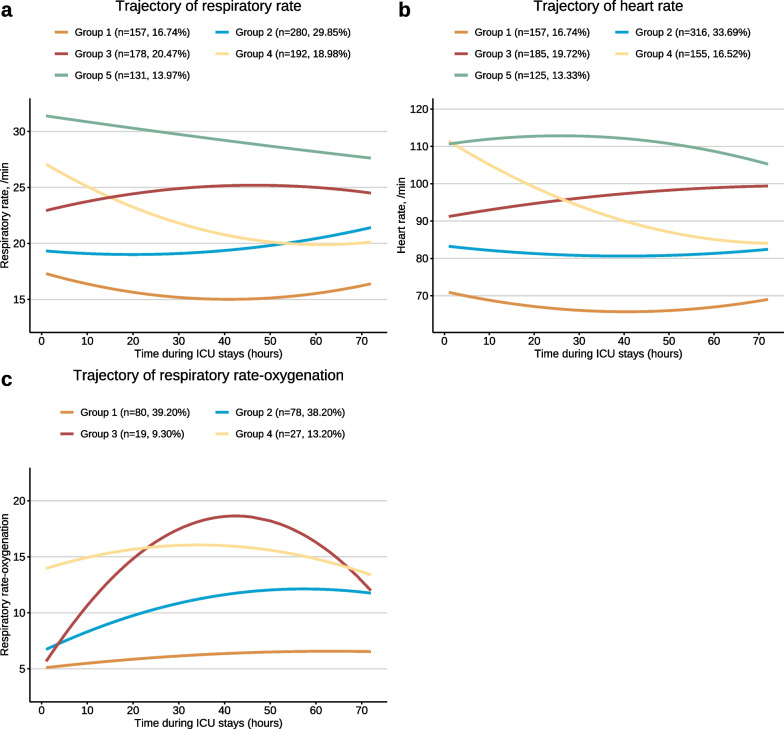
Trajectories of RR, HR, and ROX. This figure shows the distinct trajectories of respiratory rate, heart rate, and respiratory rate-oxygenation from the first 72 h of ICU stays based on data of the eICU-CRD. Using the approach of group-based trajectory model, 5 respiratory rate trajectories were presented in (**a**), 5 heart rate trajectories were presented in (**b**), 4 respiratory rate-oxygenation trajectories were presented in (**c**). *RR* respiratory rate, *HR* heart rate, *ROX* respiratory rate-oxygenation, *ICU* intensive care unit, *eICU-CRD* eICU Collaborative Research Database

Figure 1 presents the trajectories of RR, HR, and ROX. For RR curves, group 1 (n = 157, 16.74%) had a low stable level. Group 2 (n = 280, 29.85%) had low levels at baseline and then elevated. Group 3 (n = 178, 20.47%) started with a middle level, which then increased and then declined. Group 4 (n = 192, 18.98%) started with a high level of RR, which then decreased to the middle level. Group 5 (n = 131, 13.97%) had persistent high level with a slight downward trend. Group 1 was younger, and group 3 and group 5 had higher rates of mortality. For HR curves, group 1 (n = 157, 16.74%), group 4 (n = 155, 16.52%), and group 5 (n = 125, 13.33%) had similar trends compared with those of RR. The HR in group 2 (n = 316, 33.69%) had a medium–low stable level; group 3 (n = 185, 19.72%) started with a medium level, which then increased to nearly 100 beats per minute. The ROX curves of groups 1, 2, and 3 had similar initial levels. The level of group 1 (n = 80, 39.20%) slightly increased, group 2 (n = 78, 38.20%) increased to moderate level, and the level of group 3 (n = 19, 9.30%) sharply increased and declined to middle level with an inverted U-shaped curve. Group 4 (n = 27, 13.20%) had high stable ROX.

Table 2 presents the odds ratios (ORs) and 95% confidence intervals (CIs) of trajectories on the risk of 28-day mortality. In the multivariable adjustment models, compared with trajectory group 4 of RR, the ORs and 95% CIs of group 3 were 2.74 (1.48–5.07) (*P* = 0.001). Compared with trajectory group 1 of HR, the ORs and 95% CIs were 2.20 (1.19–4.08) (*P* = 0.012) for group 2, 2.70 (1.40–5.23) (*P* = 0.003) for group 3, and 2.16 (1.04–4.49) (*P* = 0.040) for group 5. There was no significant difference in mortality risk among trajectory groups of ROX.

**Table 2:** ORs and 95% CIs of trajectories on risk of 28-day mortality

Trajectory group	Logistic models			
RR	Univariate model	*P*	Multivariate model	*P*
4	Reference	–	Reference	–
1	1.38 (0.78–2.46)	0.272	2.33 (1.16–4.70)	0.018
2	1.24 (0.74–2.08)	0.415	1.59 (0.85–2.98)	0.143
3	2.17 (1.29–3.67)	0.004	2.74 (1.48–5.07)	0.001
5	1.97 (1.11–3.49)	0.021	1.60 (0.82–3.12)	0.169

HR	Univariate model	*P*	Multivariate model	*P*
1	Reference	–	Reference	–
2	1.99(1.15–3.45)	0.014	2.20(1.19–4.08)	0.012
3	2.54(1.42–4.55)	0.002	2.70(1.40–5.23)	0.003
4	1.33(0.70–2.54)	0.387	1.17(0.57–2.41)	0.675
5	2.40(1.28–4.49)	0.006	2.16(1.04–4.49)	0.040

ROX	Univariate model	*P*	Multivariate model	*P*
3	Reference	–	Reference	–
1	4.58 (0.99–21.25)	0.052	2.37 (0.44–12.87)	0.317
2	3.13 (0.67–14.73)	0.148	2.01 (0.38–10.73)	0.414
4	2.43 (0.43–13.61)	0.313	1.85 (0.29–11.90)	0.516

Trajectory groups with lowest rate of 28-day mortality were considered as the reference in the logistic model. In univariate model, current trajectory group was adjusted. In multivariable model, current trajectory group, age, sex, SOFA, APS-III, vasopressin, ventilation, WBCs, BUN, creatinine, and respiratory rate were adjusted

*ORs* odds ratios, *CIs* confidence intervals, *SOFA* Sequential Organ Failure Assessment, *APS-III* Acute Physiology Score III, *WBCs* white blood cells, *BUN* blood urea nitrogen, *RR* respiratory rate, *HR* heart rate, *ROX* respiratory rate-oxygenation

**Fig. 2:**
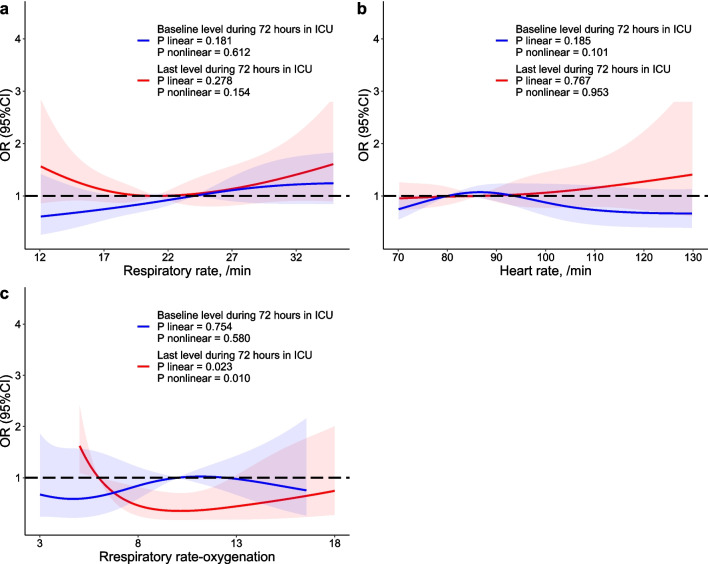
RCS of baseline, post-treatment levels of RR, HR, and ROX with 28-day mortality. Blue lines represent associations of baseline RR, heart rate, and ROX with 28-day mortality whereas red lines represent associations of post-treatment RR, heart rate, and ROX with 28-day mortality. For RR, 99.89% baseline levels were observed during 1 to 24 h in the ICU whereas 99.57% last levels were observed during 25 to 72 h in the ICU; for heart rate, 99.89% baseline levels were observed during 1 to 24 h in the ICU whereas 100.00% last levels were observed during 25 to 72 h in the ICU; for ROX, 100.00% baseline levels were observed during 1 to 24 h in the ICU whereas 97.55% levels were observed during 25 to 72 h in the ICU. *RCS* restricted cubic splines, *ORs* odds ratios, *RR* respiratory rate, *HR* heart rate, *ROX* respiratory rate-oxygenation, *ICU* intensive care unit

Figure 2 presents restricted cubic splines (RCS) to model relationships of the baseline and last levels of RR, HR, and ROX with 28-day mortality. The cubic spline function revealed no linear or nonlinear effect of baseline levels of RR (*P* for linear = 0.181, *P* for nonlinear = 0.612), HR (*P* for linear = 0.185, *P* for nonlinear = 0.101), or ROX (*P* for linear = 0.754, *P* for nonlinear = 0.580) on 28-day mortality. For the dimension of last levels, RR and HR were not found to be independently associated with 28-day mortality, whereas the last level of ROX was linearly (*P* = 0.023) and nonlinearly (*P* = 0.010) associated with 28-day mortality. A lower last level of ROX indicated a higher risk of mortality.

**Fig. 3:**
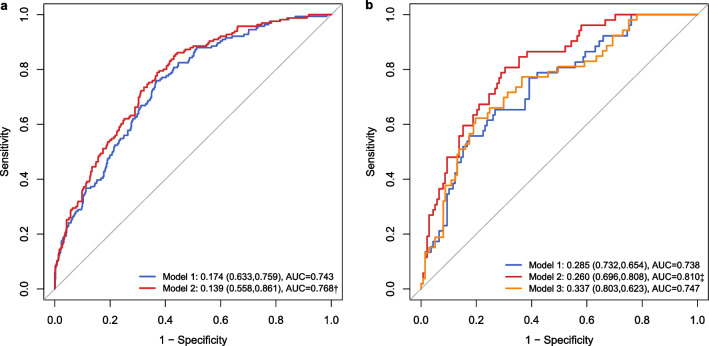
ROCs of different logistic models to predict 28-day mortality. ROCs of different logistic models in 938 population were presented in (**a**), ROCs of different logistic models in 204 population with at least 4 records of ROX were presented in (**b**). Model 1 adjusted age, sex, SOFA, APS-III, vasopressin, ventilation, WBCs, BUN, creatinine, and RR; model 2 adjusted variables in model 1 plus trajectories of RR and HR; model 3 adjusted variables in model 1 plus last measurement of ROX. *ROC* receiver operating curve, *SOFA* Sequential Organ Failure Assessment, *APS-III* Acute Physiology Score III; *WBCs* white blood cells, *BUN* blood urea nitrogen, *RR* respiratory rate, *HR* heart rate, *ROX* respiratory rate-oxygenation. †*P* = 0.020, ‡*P* = 0.015

Figure 3 presents the ROCs of the different logistic models to predict 28-day mortality. In 938 elderly individuals, the AUC of the model with adjustment for the trajectories of RR and HR was superior to that of the model without adjustment for the trajectory factors (0.768 vs. 0.743, *P* = 0.020). In 204 elderly patients with at least 4 records of ROX, the AUC of model 2 was superior to that of model 1 (0.810 vs. 0.738, *P* = 0.015), and there was no significant difference between model 1 and model 3 (0.738 vs. 0.747,* P* = 0.878).

## Discussion

We determined the shapes and numbers of the clinical featured trajectories using RR, HR, and the composite index of ROX in elderly, critically ill patients with ARDS, using data from the eICU-CRD cohort. Based on the trajectory analysis, potential subphenotypes of ARDS were identified. RR, HR, and ROX had distinct manifestations of mortality risk. For RR and HR, their longitudinal subphenotypes are risk factors for 28-day mortality and could improve the predictive ability. For the ROX index, its last levels during the initial 72 h of ICU admission are associated with 28-day mortality. To our knowledge, this is the first study to explore dynamic subphenotypes and static levels of RR, HR, and ROX, and to assess their associations with death in elderly ARDS patients who were admitted to the ICU. These findings indicate that dynamic subphenotypes of RR and HR, and static ROX after initial critical care could suggest a prognosis that may need to be managed in critically ill elderly patients with ARDS.

Compared with single time-point measurements, trajectories or average patterns of RR and HR may capture a combination of disease statuses, including dysfunctional central respiratory control, respiratory or metabolic impairments [30], infection inflammation, lung injury, and myocardial infarct. Most elderly individuals had poor RR status, among which tachypnea is usually caused by ARDS. Notably, the initial moderate level and rising stage of RR (group 3) were integral contributing factors for mortality. For HR subphenotypes, the cumulative effect of varying degrees of higher HR without a clear downward trend predicted an unfavorable prognosis. An increased HR indicates that a person has a low oxygen level, which represents a more severe condition of ARDS. Moreover, ICU patients commonly suffer from impaired physiological homeostasis and circadian rhythm disorders [31, 32], which affect neural regulation and result in HR variation [18, 33]. Furthermore, a prolonged elevated HR in critically ill, cardiac high-risk patients could result in major cardiac events [34], which may cause adverse prognosis. It needs to be noted that the lowest RR (group 1) was not independently associated with mortality which is different from that of HR. The possible reason may be that RR in group 1 was effectively controlled by clinical interventions such as mechanical ventilation, sedation, and analgesia, which dissimulate the symptoms of ventilation-induced [10] and ARDS-induced lung injury. The potential mechanism needs to be explored in the future.

The subphenotypes of ROX were not associated with mortality in elderly patients with ARDS, and interestingly, ICU inpatients could switch between two levels (low and high) of ROX. Treatment in the ICU seemed to affect ROX, as 3 subphenotypes changed to higher levels over time. Piryani et al. [16] showed that patients with a high ROX had a lower risk of mortality for ARDS, and Roca et al. [14, 15] indicated that ROX is a prognostic factor for nasal high-flow therapy. All these studies focused on the ROX at a single time point; perhaps the longitudinal measurement of ROX was not a sensitive predictor of mortality. Another possible interpretation is that the GBTM did not fit a longitudinal ROX subphenotype with a lower level, which may have a higher risk of mortality. The fitted GBTM of ROX needs to be explored in further studies.

We applied restricted cubic spline to model associations of baseline and last levels of RR-related indicators during the first 72 h of stays in the ICU with 28-day mortality, and only the last ROX was linearly and nonlinearly associated with mortality. Strauß et al. [35] indicated that compared with an RR on hospital admission of 12–20/min, an RR of 27–33/min and above 33/min were associated with high mortality in patients with community-acquired pneumonia. Our different results could be attributed to population heterogeneity and unstable changes in RR in ICU patients with ARDS. In addition, the RR may be improved by ventilation and other therapeutic interventions which are relatively homogenous behaviors in the ICU. Therefore, using single time-point measurements may not effectively differentiate between varying mortality risks. Laskey et al. [36] showed that HR at discharge in patients with heart failure is associated with mortality. Wang et al. [37] indicated that a low minimum HR under 60 bpm may be associated with a higher risk for 30-day mortality in critically ill myocardial infarction patients. The association between HR and mortality in ARDS patients is rarely explored; based on our results, the original and last HR is not related to mortality. Guo et al. [18] demonstrated a U-shaped curve relating HR fluctuation (maximum HR minus the minimum HR in the initial 24 h) to mortality in critically ill ICU patients, highlighting the implication of longitudinal HR. Our results could explain the adverse prognosis of low HR fluctuations [18], for example, sustained moderate and sustained high heart rates (groups 2 and 5) had low heart rate fluctuations, which resulted in a higher mortality risk. What is interesting is the linear and nonlinear effects of last ROX. ROX could reflect dyspnea and the severity of respiratory failure [16]. Leszek et al. [38] indicated that early measurement of the ROX index in the intermediary care unit is associated with mortality in intubated COVID-19 patients. Lee et al. [39] showed that the ROX index could be used as a prognostic marker in sepsis. We specifically investigated the last ROXs, which were measured mostly (97.55%) from 25 to 72 h in the ICU. The reinvention ability of ROX through ICU care had a specific effect on mortality. The work of Piryani et al. [16] was consistent with our findings to some extent. The possible reason for the lack of a significant risk of the longitudinal ROX subphenotype may be that high variation in ROX and transient low ROX (such as values < 5) were difficult to quantify by the trajectory model.

Dynamic subphenotypes of RR and HR had additional predictive value of 28-day mortality (AUC increased by 2.5%, *P* = 0.020), whereas last ROX during the first 72 h of ICU stays did not. This phenomenon indicats that pieces of information on longitudinal RR and HR could supplement the cause of death in addition to the portion explained by other adjusted variables. First, in critically ill elderly patients with ARDS, the dynamic RR and HR may be sensitive and early altered bioindicators of a combination of disease statuses. Second, the suppressed respiratory central and insufficient blood supply caused by persistent deterioration of RR and HR may directly result in death. Further research is needed to explore the mechanism.

There were several limitations in our study. First, trajectory analysis is a data-driven method and may not be applicable for emergencies, in which the patient dies in a shorter time. Second, the results may not apply to ICUs elsewhere with different resources that have heterogeneity in populations, environments, and treatment methods.

## Conclusion

Longitudinal dynamic subphenotypes of RR and HR and last static levels of ROX during the first 72 h of ICU stays play specific roles in 28-day mortality. These findings indicate that the dynamic subphenotypes of RR and HR, and static ROX after initial critical care could suggest a prognosis that may need to be controlled in critically ill elderly patients with ARDS.

### Supplementary Information

**Additional file1: Fig. S1.** Flow diagram used for selection of the population. **Table S1.** The model fitting process of trajectories of respiratory rate, heart rate, and respiratory rate-oxygenation. **Table S2.** Description of baseline variables for ICU patients grouped by respiratory rate trajectories. **Table S3.** Description of baseline variables for ICU patients grouped by heart rate trajectories. **Table S4.** Description of baseline variables for ICU patients grouped by respiratory rate-oxygenation trajectories.

## Data Availability

The data from the eICU-CRD can be obtained after approval of proposal with a signed data access agreement by checking physionet (https://physionet.org/).

## Abbreviations

ARDS: Acute respiratory distress syndrome
ICUs: Intensive care units
RR: Respiratory rate
CO_2_: Carbon dioxide
HR: Heart rate
PaO_2_: Oxygen partial pressure
FiO_2_: Fractional oxygen
ROX: Respiratory rate-oxygenation
SpO_2_: Pulse oxygen saturation
eICU-CRD: eICU Collaborative Research Database
ROC: Receiver operating curve
AUC: Area under the curve
ICD-9-CM: International Classification of Diseases, Ninth Revision, Clinical Modification
ICD-10-CM: International Classification of Diseases, Tenth Revision, Clinical Modification
SOFA: Sequential Organ Failure Assessment
APS-III: Acute Physiology Score III
GCS: Glasgow Coma Scale
WBCs: White blood cells
INR: International normalized ratio
PTT: Partial thromboplastin time
BUN: Blood urea nitrogen
CVD: Cerebrovascular disease
COPD: Chronic pulmonary disease
SD: Standard deviation
GBTM: Group-based trajectory model
BIC: Bayesian information criterion
ORs: Odds ratios
CIs: Confidence intervals
RCS: Restricted cubic splines
