# Supplementary material for: Identifying vital sign trajectories to predict 28-day mortality of critically ill elderly patients with acute respiratory distress syndrome
Source: Respir Res. 2024 Jan 4;25:8. doi: 10.1186/s12931-023-02643-8 (PMC10765902; doi:10.1186/s12931-023-02643-8)
Supplement: Supplementary file 1 — Additional file1: Fig. S1. Flow diagram used for selection of the population. Table S1. The model fitting process of trajectories of respiratory rate, heart rate, and respiratory rate-oxygenation. Table S2. Description of baseline variables for ICU patients grouped by respiratory rate trajectories. Table S3. Description of baseline variables for ICU patients grouped by heart rate trajectories. Table S4. Description of baseline variables for ICU patients grouped by respiratory rate-oxygenation trajectories. [file 12931_2023_2643_MOESM1_ESM.docx]

**Additional file 1**

Fig. S1 Flow diagram used for selection of the population.

Table S1 The model fitting process of trajectories of respiratory rate, heart rate, and respiratory rate-oxygenation.

Table S2 Description of baseline variables for ICU patients grouped by respiratory rate trajectories.

Table S3 Description of baseline variables for ICU patients grouped by heart rate trajectories.

Table S4 Description of baseline variables for ICU patients grouped by respiratory rate-oxygenation trajectories.


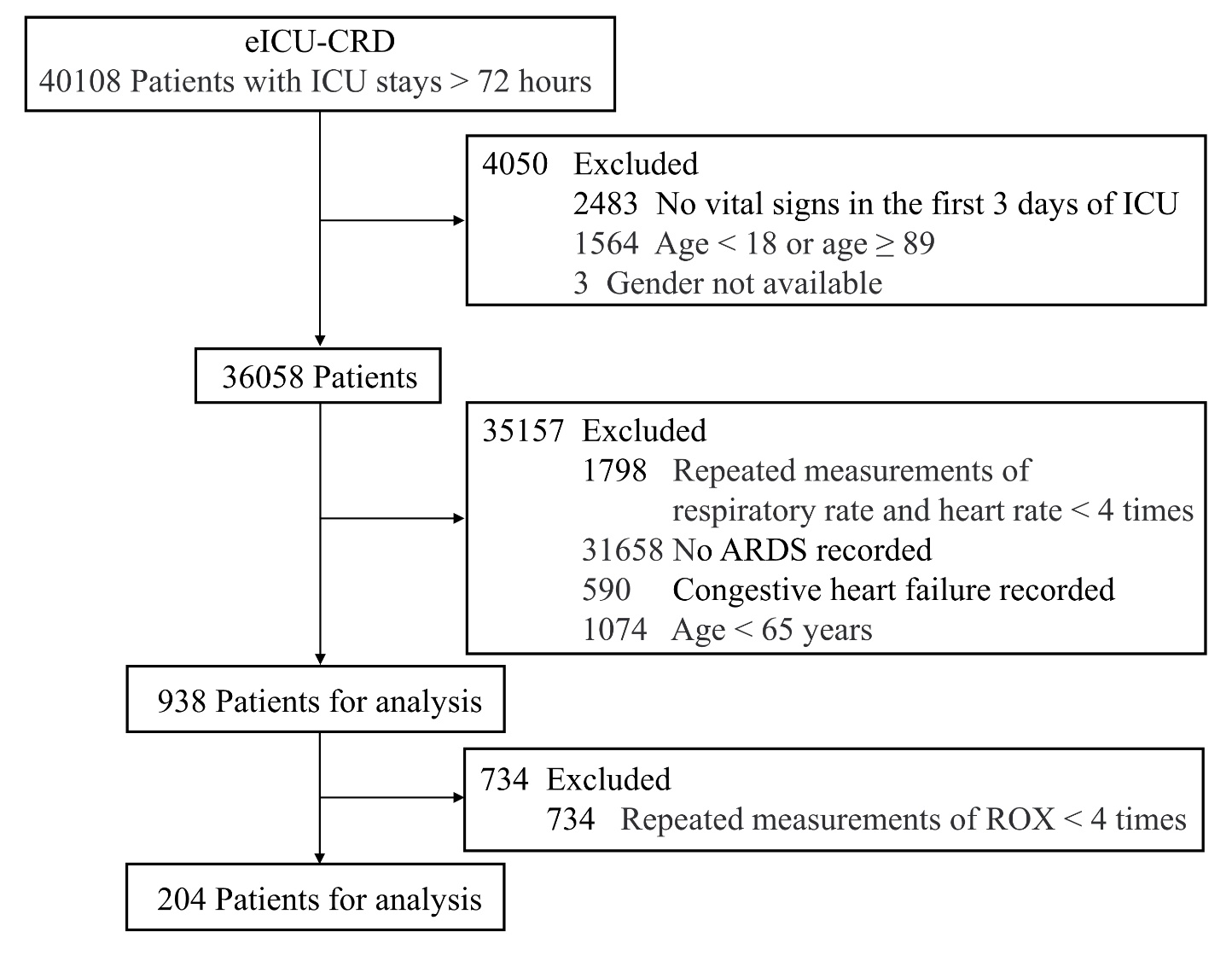


**Fig. S1 Flow diagram used for selection of the population**

**Table S1 The model fitting process of trajectories of respiratory rate, heart rate, and respiratory rate-oxygenation.**

| Trajectory variable | Number of groups | Highest order of trajectory curve | BIC | Proportion of patients | Mean posterior probabilities |
| --- | --- | --- | --- | --- | --- |
| Respiratory rate | 2 | 2/2 | -156,321.5 | 60.34%/39.66% | 98.83%/97.61% |
|  | 3 | 2/2/2 | -153,663.2 | 30.17%/47.65%/22.17% | 97.89%/96.62%/97.88% |
|  | 4 | 2/2/2/2 | -152,648.0 | 20.15%/40.09%/26.97%/12.79% | 96.44%/95.23%/94.11%/96.46% |
|  | **5** | **2/2/2/2/2** | **-151,947.3** | **16.74%/29.85%/20.47%/**  **18.98%/13.97%** | **95.05%/91.70%/93.59%/**  **91.33%/96.31%** |
| Heart rate | 2 | 2/2 | -216,012.1 | 51.71%/48.29% | 99.18%/98.89% |
|  | 3 | 2/2/2 | -212,122.0 | 33.26%/43.07%/23.67% | 98.05%/97.98%/99.05% |
|  | 4 | 2/2/2/2 | -210,170.5 | 16.63%/34.01%/33.80%/15.57% | 97.58%/97.90%/97.15%/98.23% |
|  | **5** | **2/2/2/2/2** | **-208,845.6** | **16.74%/33.69%/19.72%/**  **16.52%/13.33%** | **97.60%/97.92%/96.08%/**  **94.63%/97.33%** |
| Respiratory rate-oxygenation | 2 | 2/2 | -3,412.6 | 64.71%/35.29% | 94.97%/94.33% |
|  | 3 | 2/2/2 | -3,384.6 | 56.37%/28.92%/14.71% | 94.37%/82.95%/89.57% |
|  | **4** | **2/2/2/2** | **-3,378.2** | **39.22%/38.24%/9.31%/13.24%** | **90.67%/81.82%/84.19%/91.88%** |
|  | 5 | 2/2/2/2/2 | -3,376.0 | 39.22%/27.45%/14.22%/  17.16%/1.96% | 88.09%/80.58%/80.09%/  88.13%/95.75% |

BIC indicates Bayesian information criterion; the optimal model is highlighted in bold.

**Table S2 Description of baseline variables for ICU patients grouped by respiratory rate trajectories.**

| Variables | Group 1 (n=157) | Group 2 (n=280) | Group 3 (n=192) | Group 4 (n=178) | Group 5 (n=131) | *P* |
| --- | --- | --- | --- | --- | --- | --- |
| **Demographic information** |  |  |  |  |  |  |
| Age, year | 71.00 [68.00, 78.00] | 74.00 [70.00, 81.00] | 74.50 [69.00, 80.00] | 76.00 [71.00, 81.00] | 74.00 [69.00, 79.00] | 0.005 |
| Sex, n (%) |  |  |  |  |  | 0.715 |
| Male | 75 (47.77) | 134 (47.86) | 103 (53.65) | 91 (51.12) | 68 (51.91) |  |
| Female | 82 (52.23) | 146 (52.14) | 89 (46.35) | 87 (48.88) | 63 (48.09) |  |
| Ethnicity, n (%) |  |  |  |  |  | 0.049 |
| Caucasian | 115 (73.25) | 230 (82.14) | 157 (81.77) | 132 (74.16) | 117 (89.31) |  |
| African American | 20 (12.74) | 20 (7.14) | 9 (4.69) | 23 (12.92) | 6 (4.58) |  |
| Asian | 1 (0.64) | 2 (0.71) | 3 (1.56) | 4 (2.25) | 0 (0.00) |  |
| Hispanic | 10 (6.37) | 12 (4.29) | 10 (5.21) | 9 (5.06) | 4 (3.05) |  |
| Native American | 2 (1.27) | 2 (0.71) | 0 (0.00) | 2 (1.12) | 1 (0.76) |  |
| Other/Unknown | 9 (5.73) | 14 (5.00) | 13 (6.77) | 8 (4.49) | 3 (2.29) |  |
| First ICU location, n (%) |  |  |  |  |  | 0.019 |
| MICU | 16 (10.19) | 28 (10.00) | 25 (13.02) | 27 (15.17) | 27 (20.61) |  |
| CCU-CTICU | 12 (7.64) | 22 (7.86) | 15 (7.81) | 14 (7.87) | 10 (7.63) |  |
| NICU | 12 (7.64) | 16 (5.71) | 8 (4.17) | 7 (3.93) | 3 (2.29) |  |
| Med-Surg ICU | 80 (50.96) | 160 (57.14) | 115 (59.90) | 100 (56.18) | 78 (59.54) |  |
| Cardiac ICU | 12 (7.64) | 22 (7.86) | 12 (6.25) | 17 (9.55) | 5 (3.82) |  |
| CTICU | 5 (3.18) | 8 (2.86) | 5 (2.60) | 4 (2.25) | 0 (0.00) |  |
| SICU | 19 (12.10) | 20 (7.14) | 5 (2.60) | 8 (4.49) | 6 (4.58) |  |
| CSICU | 1 (0.64) | 4 (1.43) | 7 (3.65) | 1 (0.56) | 2 (1.53) |  |
| **Severity of illness** |  |  |  |  |  |  |
| SOFA | 6.40 (2.77) | 6.23 (2.71) | 6.30 (2.63) | 6.00 (2.70) | 6.67 (2.70) | 0.283 |
| APS-III | 52.00 [40.00, 73.00] | 51.50 [37.00, 67.75] | 50.00 [36.00, 69.25] | 52.00 [36.75, 73.00] | 59.00 [35.50, 76.00] | 0.251 |
| GCS | 11.00 [10.00, 14.00] | 13.00 [10.00, 15.00] | 13.00 [10.50, 15.00] | 14.00 [10.00, 15.00] | 13.00 [10.00, 15.00] | 0.005 |
| **Support within the first 24h** |  |  |  |  |  |  |
| Vasopressin, n (%) |  |  |  |  |  | 0.821 |
| No | 129 (82.17) | 236 (84.29) | 158 (82.29) | 146 (82.02) | 104 (79.39) |  |
| Yes | 28 (17.83) | 44 (15.71) | 34 (17.71) | 32 (17.98) | 27 (20.61) |  |
| Ventilation, n (%) |  |  |  |  |  | 0.049 |
| No | 42 (26.75) | 88 (31.43) | 49 (25.52) | 67 (37.64) | 48 (36.64) |  |
| Yes | 115 (73.25) | 192 (68.57) | 143 (74.48) | 111 (62.36) | 83 (63.36) |  |
| Dialysis, n (%) |  |  |  |  |  | 0.851 |
| No | 150 (95.54) | 272 (97.14) | 186 (96.88) | 173 (97.19) | 128 (97.71) |  |
| Yes | 7 (4.46) | 8 (2.86) | 6 (3.12) | 5 (2.81) | 3 (2.29) |  |
| **Laboratory information** |  |  |  |  |  |  |
| Hemoglobin, g/dL | 10.68 (2.45) | 10.58 (2.14) | 10.80 (2.16) | 10.48 (2.15) | 10.55 (2.05) | 0.672 |
| Platelets, 10^9^/L | 188.00 [155.00, 237.00] | 198.00 [148.00, 258.50] | 197.00 [145.75, 240.25] | 204.00 [149.00, 273.75] | 226.00 [155.00, 289.00] | 0.206 |
| WBCs, 10^9^/L | 10.90 [7.60, 15.40] | 11.63 [8.81, 16.45] | 11.65 [8.20, 16.42] | 11.22 [8.20, 15.75] | 12.85 [8.43, 19.20] | 0.174 |
| INR, % | 1.20 [1.10, 1.50] | 1.20 [1.10, 1.40] | 1.29 [1.10, 1.60] | 1.40 [1.17, 1.80] | 1.20 [1.10, 1.50] | < 0.001 |
| PTT, s | 32.00 [26.85, 39.62] | 31.90 [27.00, 38.40] | 33.30 [29.00, 42.55] | 34.00 [29.10, 42.10] | 34.50 [29.70, 43.15] | 0.041 |
| BUN, mg/dL | 24.00 [17.00, 39.00] | 28.00 [19.00, 43.25] | 28.00 [18.00, 41.25] | 25.00 [17.00, 40.00] | 25.00 [17.25, 41.00] | 0.149 |
| Creatinine, mg/dL | 1.00 [0.71, 1.78] | 1.14 [0.81, 1.71] | 1.15 [0.80, 1.77] | 1.10 [0.74, 1.66] | 1.08 [0.74, 1.94] | 0.569 |
| Sodium, mmol/L | 139.09 (5.61) | 139.11 (6.30) | 138.59 (6.51) | 137.98 (5.58) | 139.30 (6.27) | 0.203 |
| Potassium, mmol/L | 4.20 (0.74) | 4.19 (0.72) | 4.16 (0.79) | 4.21 (0.81) | 4.01 (0.61) | 0.057 |
| Calcium, mg/dL | 8.24 (0.82) | 8.22 (0.79) | 8.33 (1.07) | 8.31 (0.72) | 8.20 (1.08) | 0.648 |
| Chloride, mmol/L | 103.48 (7.22) | 103.49 (7.23) | 102.86 (7.72) | 103.94 (7.21) | 104.88 (7.85) | 0.230 |
| Glucose, mg/dL | 138.00 [108.00, 181.00] | 136.00 [111.00, 175.25] | 145.50 [115.75, 180.25] | 149.50 [117.00, 190.00] | 131.00 [105.00, 176.00] | 0.133 |
| Bicarbonate, mmol/L | 24.99 (5.95) | 26.05 (6.04) | 25.70 (5.91) | 24.05 (6.51) | 24.82 (6.70) | 0.018 |
| **Charlson comorbidity** |  |  |  |  |  |  |
| Myocardial infarct, n (%) |  |  |  |  |  | 0.041 |
| No | 157 (100.00) | 272 (97.14) | 182 (94.79) | 171 (96.07) | 129 (98.47) |  |
| Yes | 0 (0.00) | 8 (2.86) | 10 (5.21) | 7 (3.93) | 2 (1.53) |  |
| Peripheral vascular disease, n (%) |  |  |  |  |  | 0.141 |
| No | 154 (98.09) | 280 (100.00) | 189 (98.44) | 176 (98.88) | 131 (100.00) |  |
| Yes | 3 (1.91) | 0 (0.00) | 3 (1.56) | 2 (1.12) | 0 (0.00) |  |
| Cerebrovascular disease, n (%) |  |  |  |  |  | 0.002 |
| No | 141 (89.81) | 266 (95.00) | 187 (97.40) | 173 (97.19) | 129 (98.47) |  |
| Yes | 16 (10.19) | 14 (5.00) | 5 (2.60) | 5 (2.81) | 2 (1.53) |  |
| Dementia, n (%) |  |  |  |  |  | 0.108 |
| No | 150 (95.54) | 268 (95.71) | 188 (97.92) | 175 (98.31) | 122 (93.13) |  |
| Yes | 7 (4.46) | 12 (4.29) | 4 (2.08) | 3 (1.69) | 9 (6.87) |  |
| Chronic pulmonary disease, n (%) |  |  |  |  |  | 0.855 |
| No | 127 (80.89) | 228 (81.43) | 150 (78.12) | 140 (78.65) | 102 (77.86) |  |
| Yes | 30 (19.11) | 52 (18.57) | 42 (21.88) | 38 (21.35) | 29 (22.14) |  |
| Rheumatic disease, n (%) |  |  |  |  |  | 0.317 |
| No | 156 (99.36) | 280 (100.00) | 191 (99.48) | 176 (98.88) | 131 (100.00) |  |
| Yes | 1 (0.64) | 0 (0.00) | 1 (0.52) | 2 (1.12) | 0 (0.00) |  |
| Peptic ulcer disease, n (%) |  |  |  |  |  | 0.024 |
| No | 153 (97.45) | 278 (99.29) | 192 (100.00) | 178 (100.00) | 131 (100.00) |  |
| Yes | 4 (2.55) | 2 (0.71) | 0 (0.00) | 0 (0.00) | 0 (0.00) |  |
| Mild liver disease, n (%) |  |  |  |  |  | 0.955 |
| No | 153 (97.45) | 274 (97.86) | 188 (97.92) | 173 (97.19) | 129 (98.47) |  |
| Yes | 4 (2.55) | 6 (2.14) | 4 (2.08) | 5 (2.81) | 2 (1.53) |  |
| Severe liver disease, n (%) |  |  |  |  |  | 0.014 |
| No | 157 (100.00) | 280 (100.00) | 192 (100.00) | 175 (98.31) | 131 (100.00) |  |
| Yes | 0 (0.00) | 0 (0.00) | 0 (0.00) | 3 (1.69) | 0 (0.00) |  |
| Diabetes, n (%) |  |  |  |  |  | 0.691 |
| No | 141 (89.81) | 249 (88.93) | 170 (88.54) | 155 (87.08) | 111 (84.73) |  |
| Yes | 16 (10.19) | 31 (11.07) | 22 (11.46) | 23 (12.92) | 20 (15.27) |  |
| Paraplegia, n (%) |  |  |  |  |  | 0.702 |
| No | 157 (100.00) | 280 (100.00) | 191 (99.48) | 178 (100.00) | 131 (100.00) |  |
| Yes | 0 (0.00) | 0 (0.00) | 1 (0.52) | 0 (0.00) | 0 (0.00) |  |
| Renal disease, n (%) |  |  |  |  |  | 0.665 |
| No | 134 (85.35) | 250 (89.29) | 171 (89.06) | 160 (89.89) | 118 (90.08) |  |
| Yes | 23 (14.65) | 30 (10.71) | 21 (10.94) | 18 (10.11) | 13 (9.92) |  |
| Malignant cancer, n (%) |  |  |  |  |  | 0.237 |
| No | 147 (93.63) | 265 (94.64) | 173 (90.10) | 160 (89.89) | 119 (90.84) |  |
| Yes | 10 (6.37) | 15 (5.36) | 19 (9.90) | 18 (10.11) | 12 (9.16) |  |
| Metastatic solid tumor, n (%) |  |  |  |  |  | 0.528 |
| No | 157 (100.00) | 278 (99.29) | 191 (99.48) | 175 (98.31) | 130 (99.24) |  |
| Yes | 0 (0.00) | 2 (0.71) | 1 (0.52) | 3 (1.69) | 1 (0.76) |  |
| Aids, n (%) |  |  |  |  |  | > 0.999 |
| No | 157 (100.00) | 279 (99.64) | 191 (99.48) | 178 (100.00) | 131 (100.00) |  |
| Yes | 0 (0.00) | 1 (0.36) | 1 (0.52) | 0 (0.00) | 0 (0.00) |  |
| **Vital signs** |  |  |  |  |  |  |
| RR, /min | 19.54 (6.31) | 21.35 (5.92) | 25.07 (6.45) | 28.74 (6.63) | 33.27 (8.51) | < 0.001 |
| HR, /min | 90.28 (21.99) | 91.15 (20.76) | 94.33 (20.50) | 102.84 (21.72) | 107.08 (23.08) | < 0.001 |
| ROX | 8.33 [5.08, 11.89] | 8.01 [4.45, 11.62] | 5.65 [4.26, 7.53] | 4.33 [2.84, 6.45] | 5.11 [3.49, 6.66] | < 0.001 |
| **Mortality outcome** |  |  |  |  |  |  |
| 28-day mortality, n (%) |  |  |  |  |  | 0.014 |
| No | 127 (80.89) | 231 (82.50) | 140 (72.92) | 152 (85.39) | 98 (74.81) |  |
| Yes | 30 (19.11) | 49 (17.50) | 52 (27.08) | 26 (14.61) | 33 (25.19) |  |

Data are presented using mean (standard deviation (SD)), median (lower quartile-upper quartile), or number (proportion, %). ICU: intensive care unit; SOFA: Sequential Organ Failure Assessment; APS-III: Acute Physiology Score III; GCS: Glasgow Coma Scale; WBCs: white blood cells. INR: international normalized ratio; PTT: partial thromboplastin time; BUN: blood urea nitrogen; RR: respiratory rate; HR: heart rate; ROX: respiratory rate-oxygenation.

**Table S3 Description of baseline variables for ICU patients grouped by heart rate trajectories.**

| Variables | Group 1 (n=157) | Group 2 (n=316) | Group 3 (n=185) | Group 4 (n=155) | Group 5 (n=125) | *P* |
| --- | --- | --- | --- | --- | --- | --- |
| **Demographic information** |  |  |  |  |  |  |
| Age, year | 75.00 [69.00, 81.00] | 75.00 [69.00, 80.00] | 74.00 [69.00, 80.00] | 74.00 [69.00, 81.00] | 74.00 [68.00, 79.00] | 0.648 |
| Sex, n (%) |  |  |  |  |  | 0.280 |
| Male | 87 (55.41) | 159 (50.32) | 87 (47.03) | 83 (53.55) | 55 (44.00) |  |
| Female | 70 (44.59) | 157 (49.68) | 98 (52.97) | 72 (46.45) | 70 (56.00) |  |
| Ethnicity, n (%) |  |  |  |  |  | 0.975 |
| Caucasian | 123 (78.34) | 251 (79.43) | 150 (81.08) | 127 (81.94) | 100 (80.00) |  |
| African American | 17 (10.83) | 24 (7.59) | 12 (6.49) | 14 (9.03) | 11 (8.80) |  |
| Asian | 2 (1.27) | 3 (0.95) | 2 (1.08) | 2 (1.29) | 1 (0.80) |  |
| Hispanic | 4 (2.55) | 18 (5.70) | 11 (5.95) | 5 (3.23) | 7 (5.60) |  |
| Native American | 2 (1.27) | 4 (1.27) | 1 (0.54) | 0 (0.00) | 0 (0.00) |  |
| Other/Unknown | 9 (5.73) | 16 (5.06) | 9 (4.86) | 7 (4.52) | 6 (4.80) |  |
| First ICU location, n (%) |  |  |  |  |  | 0.013 |
| MICU | 16 (10.19) | 38 (12.03) | 27 (14.59) | 25 (16.13) | 17 (13.60) |  |
| CCU-CTICU | 10 (6.37) | 24 (7.59) | 18 (9.73) | 13 (8.39) | 8 (6.40) |  |
| NICU | 13 (8.28) | 19 (6.01) | 11 (5.95) | 2 (1.29) | 1 (0.80) |  |
| Med-Surg ICU | 85 (54.14) | 180 (56.96) | 90 (48.65) | 95 (61.29) | 83 (66.40) |  |
| Cardiac ICU | 19 (12.10) | 22 (6.96) | 14 (7.57) | 8 (5.16) | 5 (4.00) |  |
| CTICU | 2 (1.27) | 14 (4.43) | 5 (2.70) | 1 (0.65) | 0 (0.00) |  |
| SICU | 10 (6.37) | 14 (4.43) | 17 (9.19) | 9 (5.81) | 8 (6.40) |  |
| CSICU | 2 (1.27) | 5 (1.58) | 3 (1.62) | 2 (1.29) | 3 (2.40) |  |
| **Severity of illness** |  |  |  |  |  |  |
| SOFA | 6.31 (2.59) | 6.26 (2.69) | 6.24 (2.57) | 6.37 (2.81) | 6.32 (2.94) | 0.993 |
| APS-III | 48.50 [38.25, 70.00] | 50.00 [34.00, 69.25] | 54.00 [36.00, 67.50] | 56.50 [37.50, 72.00] | 57.00 [41.00, 80.00] | 0.035 |
| GCS | 12.50 [10.00, 15.00] | 13.00 [10.00, 15.00] | 13.00 [10.00, 15.00] | 13.00 [10.00, 15.00] | 12.50 [10.00, 14.00] | 0.738 |
| **Support within the first 24h** |  |  |  |  |  |  |
| Vasopressin, n (%) |  |  |  |  |  | 0.408 |
| No | 131 (83.44) | 265 (83.86) | 150 (81.08) | 131 (84.52) | 96 (76.80) |  |
| Yes | 26 (16.56) | 51 (16.14) | 35 (18.92) | 24 (15.48) | 29 (23.20) |  |
| Ventilation, n (%) |  |  |  |  |  | 0.034 |
| No | 41 (26.11) | 92 (29.11) | 62 (33.51) | 46 (29.68) | 53 (42.40) |  |
| Yes | 116 (73.89) | 224 (70.89) | 123 (66.49) | 109 (70.32) | 72 (57.60) |  |
| Dialysis, n (%) |  |  |  |  |  | 0.445 |
| No | 150 (95.54) | 304 (96.20) | 181 (97.84) | 153 (98.71) | 121 (96.80) |  |
| Yes | 7 (4.46) | 12 (3.80) | 4 (2.16) | 2 (1.29) | 4 (3.20) |  |
| **Laboratory information** |  |  |  |  |  |  |
| Hemoglobin, g/dL | 10.76 (2.25) | 10.63 (2.16) | 10.51 (2.06) | 10.71 (2.22) | 10.45 (2.32) | 0.720 |
| Platelets, 10^9^/L | 182.00 [144.00, 249.00] | 195.00 [149.00, 259.00] | 214.50 [155.75, 273.25] | 199.00 [152.50, 252.00] | 209.00 [132.00, 280.00] | 0.458 |
| WBCs, 10^9^/L | 10.67 [7.84, 14.80] | 11.40 [8.10, 15.15] | 13.12 [ 8.88, 18.62] | 11.79 [8.55, 16.45] | 12.30 [7.40, 19.10] | 0.047 |
| INR, % | 1.20 [1.10, 1.40] | 1.20 [1.10, 1.55] | 1.20 [1.10, 1.60] | 1.30 [1.10, 1.65] | 1.32 [1.16, 1.70] | 0.117 |
| PTT, s | 31.40 [27.37, 39.05] | 32.10 [27.75, 40.20] | 33.90 [27.48, 42.12] | 33.60 [28.90, 42.50] | 35.00 [29.10, 41.85] | 0.221 |
| BUN, mg/dL | 26.00 [19.00, 41.00] | 26.00 [16.50, 40.00] | 29.00 [18.00, 45.00] | 25.00 [17.00, 40.50] | 25.00 [17.00, 49.00] | 0.238 |
| Creatinine, mg/dL | 1.20 [0.81, 1.79] | 1.08 [0.76, 1.68] | 1.18 [0.78, 1.90] | 0.97 [0.71, 1.42] | 1.23 [0.80, 2.04] | 0.032 |
| Sodium, mmol/L | 139.45 (5.97) | 138.86 (5.42) | 138.20 (7.16) | 138.93 (5.97) | 138.65 (6.36) | 0.522 |
| Potassium, mmol/L | 4.24 (0.71) | 4.13 (0.74) | 4.20 (0.77) | 4.16 (0.79) | 4.10 (0.70) | 0.384 |
| Calcium, mg/dL | 8.37 (0.73) | 8.29 (0.89) | 8.27 (0.75) | 8.17 (0.76) | 8.15 (1.30) | 0.150 |
| Chloride, mmol/L | 103.61 (7.17) | 103.53 (7.00) | 102.86 (8.03) | 104.06 (7.61) | 104.58 (7.56) | 0.379 |
| Glucose, mg/dL | 144.00 [110.00, 176.00] | 137.00 [109.00, 179.00] | 144.00 [113.00, 181.00] | 142.00 [118.50, 187.50] | 140.00 [110.00, 181.00] | 0.577 |
| Bicarbonate, mmol/L | 26.17 (6.35) | 25.47 (5.77) | 25.13 (6.24) | 25.40 (6.64) | 23.54 (6.27) | 0.017 |
| **Charlson comorbidity** |  |  |  |  |  |  |
| Myocardial infarct, n (%) |  |  |  |  |  | 0.668 |
| No | 152 (96.82) | 309 (97.78) | 178 (96.22) | 149 (96.13) | 123 (98.40) |  |
| Yes | 5 (3.18) | 7 (2.22) | 7 (3.78) | 6 (3.87) | 2 (1.60) |  |
| Peripheral vascular disease, n (%) |  |  |  |  |  | 0.956 |
| No | 155 (98.73) | 313 (99.05) | 184 (99.46) | 154 (99.35) | 124 (99.20) |  |
| Yes | 2 (1.27) | 3 (0.95) | 1 (0.54) | 1 (0.65) | 1 (0.80) |  |
| Cerebrovascular disease, n (%) |  |  |  |  |  | 0.088 |
| No | 145 (92.36) | 299 (94.62) | 178 (96.22) | 152 (98.06) | 122 (97.60) |  |
| Yes | 12 (7.64) | 17 (5.38) | 7 (3.78) | 3 (1.94) | 3 (2.40) |  |
| Dementia, n (%) |  |  |  |  |  | 0.160 |
| No | 150 (95.54) | 311 (98.42) | 177 (95.68) | 147 (94.84) | 118 (94.40) |  |
| Yes | 7 (4.46) | 5 (1.58) | 8 (4.32) | 8 (5.16) | 7 (5.60) |  |
| Chronic pulmonary disease, n (%) |  |  |  |  |  | 0.598 |
| No | 132 (84.08) | 252 (79.75) | 144 (77.84) | 120 (77.42) | 99 (79.20) |  |
| Yes | 25 (15.92) | 64 (20.25) | 41 (22.16) | 35 (22.58) | 26 (20.80) |  |
| Rheumatic disease, n (%) |  |  |  |  |  | 0.216 |
| No | 157 (100.00) | 315 (99.68) | 185 (100.00) | 154 (99.35) | 123 (98.40) |  |
| Yes | 0 (0.00) | 1 (0.32) | 0 (0.00) | 1 (0.65) | 2 (1.60) |  |
| Peptic ulcer disease, n (%) |  |  |  |  |  | 0.435 |
| No | 157 (100.00) | 312 (98.73) | 184 (99.46) | 154 (99.35) | 125 (100.00) |  |
| Yes | 0 (0.00) | 4 (1.27) | 1 (0.54) | 1 (0.65) | 0 (0.00) |  |
| Mild liver disease, n (%) |  |  |  |  |  | 0.281 |
| No | 155 (98.73) | 309 (97.78) | 181 (97.84) | 153 (98.71) | 119 (95.20) |  |
| Yes | 2 (1.27) | 7 (2.22) | 4 (2.16) | 2 (1.29) | 6 (4.80) |  |
| Severe liver disease, n (%) |  |  |  |  |  | 0.617 |
| No | 157 (100.00) | 314 (99.37) | 184 (99.46) | 155 (100.00) | 125 (100.00) |  |
| Yes | 0 (0.00) | 2 (0.63) | 1 (0.54) | 0 (0.00) | 0 (0.00) |  |
| Diabetes, n (%) |  |  |  |  |  | 0.750 |
| No | 136 (86.62) | 274 (86.71) | 167 (90.27) | 138 (89.03) | 111 (88.80) |  |
| Yes | 21 (13.38) | 42 (13.29) | 18 (9.73) | 17 (10.97) | 14 (11.20) |  |
| Paraplegia, n (%) |  |  |  |  |  | 0.289 |
| No | 156 (99.36) | 316 (100.00) | 185 (100.00) | 155 (100.00) | 125 (100.00) |  |
| Yes | 1 (0.64) | 0 (0.00) | 0 (0.00) | 0 (0.00) | 0 (0.00) |  |
| Renal disease, n (%) |  |  |  |  |  | 0.700 |
| No | 141 (89.81) | 278 (87.97) | 161 (87.03) | 142 (91.61) | 111 (88.80) |  |
| Yes | 16 (10.19) | 38 (12.03) | 24 (12.97) | 13 (8.39) | 14 (11.20) |  |
| Malignant cancer, n (%) |  |  |  |  |  | 0.429 |
| No | 150 (95.54) | 292 (92.41) | 168 (90.81) | 140 (90.32) | 114 (91.20) |  |
| Yes | 7 (4.46) | 24 (7.59) | 17 (9.19) | 15 (9.68) | 11 (8.80) |  |
| Metastatic solid tumor, n (%) |  |  |  |  |  | 0.039 |
| No | 156 (99.36) | 316 (100.00) | 182 (98.38) | 155 (100.00) | 122 (97.60) |  |
| Yes | 1 (0.64) | 0 (0.00) | 3 (1.62) | 0 (0.00) | 3 (2.40) |  |
| Aids, n (%) |  |  |  |  |  | 0.518 |
| No | 157 (100.00) | 315 (99.68) | 185 (100.00) | 155 (100.00) | 124 (99.20) |  |
| Yes | 0 (0.00) | 1 (0.32) | 0 (0.00) | 0 (0.00) | 1 (0.80) |  |
| **Vital signs** |  |  |  |  |  |  |
| RR, /min | 23.36 (8.09) | 23.50 (7.52) | 25.42 (8.47) | 26.75 (7.45) | 27.14 (8.50) | < 0.001 |
| HR, /min | 74.73 (16.80) | 89.03 (16.20) | 97.26 (16.13) | 115.39 (18.80) | 115.18 (19.11) | < 0.001 |
| ROX | 5.85 [4.17, 9.56] | 6.79 [4.55, 10.10] | 6.79 [4.26, 9.57] | 4.80 [3.55, 8.39] | 4.26 [3.49, 6.90] | 0.069 |
| **Mortality outcome** |  |  |  |  |  |  |
| 28-day mortality, n (%) |  |  |  |  |  | 0.006 |
| No | 138 (87.90) | 248 (78.48) | 137 (74.05) | 131 (84.52) | 94 (75.20) |  |
| Yes | 19 (12.10) | 68 (21.52) | 48 (25.95) | 24 (15.48) | 31 (24.80) |  |

Data are presented using mean (standard deviation (SD)), median (lower quartile-upper quartile), or number (proportion, %). ICU: intensive care unit; SOFA: Sequential Organ Failure Assessment; APS-III: Acute Physiology Score III; GCS: Glasgow Coma Scale; WBCs: white blood cells. INR: international normalized ratio; PTT: partial thromboplastin time; BUN: blood urea nitrogen; RR: respiratory rate; HR: heart rate; ROX: respiratory rate-oxygenation.

**Table S4 Description of baseline variables for ICU patients grouped by respiratory rate-oxygenation trajectories.**

| Variables | Group 1 (n=80) | Group 2 (n=78) | Group 3 (n=19) | Group 4 (n=27) | *P* |
| --- | --- | --- | --- | --- | --- |
| **Demographic information** |  |  |  |  |  |
| Age, year | 73.00 [69.00, 79.00] | 74.00 [68.25, 79.75] | 71.00 [67.00, 78.50] | 71.00 [69.00, 79.50] | 0.910 |
| Sex, n (%) |  |  |  |  | 0.005 |
| Male | 40 (50.00) | 43 (55.13) | 2 (10.53) | 15 (55.56) |  |
| Female | 40 (50.00) | 35 (44.87) | 17 (89.47) | 12 (44.44) |  |
| Ethnicity, n (%) |  |  |  |  | 0.438 |
| Caucasian | 57 (71.25) | 55 (70.51) | 10 (52.63) | 22 (81.48) |  |
| African American | 9 (11.25) | 9 (11.54) | 3 (15.79) | 3 (11.11) |  |
| Asian | 1 (1.25) | 0 (0.00) | 0 (0.00) | 0 (0.00) |  |
| Hispanic | 11 (13.75) | 8 (10.26) | 4 (21.05) | 1 (3.70) |  |
| Native American | 1 (1.25) | 4 (5.13) | 0 (0.00) | 1 (3.70) |  |
| Other/Unknown | 1 (1.25) | 2 (2.56) | 2 (10.53) | 0 (0.00) |  |
| First ICU location, n (%) |  |  |  |  | < 0.001 |
| MICU | 10 (12.50) | 13 (16.67) | 2 (10.53) | 3 (11.11) |  |
| CCU-CTICU | 6 (7.50) | 1 (1.28) | 1 (5.26) | 5 (18.52) |  |
| NICU | 3 (3.75) | 1 (1.28) | 5 (26.32) | 8 (29.63) |  |
| Med-Surg ICU | 46 (57.50) | 54 (69.23) | 8 (42.11) | 3 (11.11) |  |
| Cardiac ICU | 5 (6.25) | 4 (5.13) | 2 (10.53) | 1 (3.70) |  |
| CTICU | 2 (2.50) | 3 (3.85) | 0 (0.00) | 2 (7.41) |  |
| SICU | 3 (3.75) | 1 (1.28) | 1 (5.26) | 2 (7.41) |  |
| CSICU | 5 (6.25) | 1 (1.28) | 0 (0.00) | 3 (11.11) |  |
| **Severity of illness** |  |  |  |  |  |
| SOFA | 8.55 (2.08) | 7.90 (2.46) | 6.95 (2.27) | 7.44 (2.61) | 0.020 |
| APS-III | 69.00 [58.75, 91.00] | 65.00 [52.25, 81.25] | 63.00 [49.00, 80.00] | 59.00 [39.00, 71.00] | 0.151 |
| GCS | 11.00 [10.00, 14.00] | 11.00 [10.00, 14.00] | 10.00 [10.00, 12.50] | 10.00 [10.00, 11.00] | 0.266 |
| **Support within the first 24h** |  |  |  |  |  |
| Vasopressin, n (%) |  |  |  |  | 0.568 |
| No | 56 (70.00) | 58 (74.36) | 11 (57.89) | 19 (70.37) |  |
| Yes | 24 (30.00) | 20 (25.64) | 8 (42.11) | 8 (29.63) |  |
| Ventilation, n (%) |  |  |  |  | 0.073 |
| No | 17 (21.25) | 12 (15.38) | 0 (0.00) | 2 (7.41) |  |
| Yes | 63 (78.75) | 66 (84.62) | 19 (100.00) | 25 (92.59) |  |
| Dialysis, n (%) |  |  |  |  | 0.373 |
| No | 77 (96.25) | 74 (94.87) | 19 (100.00) | 24 (88.89) |  |
| Yes | 3 (3.75) | 4 (5.13) | 0 (0.00) | 3 (11.11) |  |
| **Laboratory information** |  |  |  |  |  |
| Hemoglobin, g/dL | 10.79 (2.26) | 10.68 (2.50) | 10.34 (2.43) | 10.69 (1.88) | 0.905 |
| Platelets, 10^9^/L | 190.00 [130.50, 259.75] | 180.50 [144.00, 232.50] | 193.00 [137.00, 345.50] | 189.00 [149.50, 255.00] | 0.909 |
| WBCs, 10^9^/L | 12.55 [8.70, 19.33] | 11.00 [8.64, 15.97] | 10.50 [7.95, 15.97] | 10.50 [6.40, 14.55] | 0.564 |
| INR, % | 1.20 [1.10, 1.50] | 1.20 [1.07, 1.63] | 1.10 [1.10, 1.30] | 1.30 [1.07, 1.57] | 0.534 |
| PTT, s | 32.30 [28.90, 39.00] | 32.80 [29.00, 43.20] | 32.60 [28.55, 40.10] | 33.00 [27.00, 48.00] | 0.930 |
| BUN, mg/dL | 25.00 [18.00, 42.00] | 27.00 [20.00, 41.50] | 22.00 [16.00, 26.50] | 32.00 [18.00, 51.50] | 0.169 |
| Creatinine, mg/dL | 1.13 [0.79, 1.82] | 1.18 [0.80, 1.86] | 0.84 [0.75, 1.17] | 1.22 [0.83, 2.16] | 0.247 |
| Sodium, mmol/L | 139.30 (5.73) | 139.39 (6.05) | 139.47 (6.02) | 138.63 (7.40) | 0.969 |
| Potassium, mmol/L | 4.06 (0.67) | 4.29 (0.90) | 3.98 (0.81) | 4.52 (0.70) | 0.018 |
| Calcium, mg/dL | 8.05 (0.79) | 8.10 (0.85) | 7.85 (0.61) | 8.02 (0.84) | 0.543 |
| Chloride, mmol/L | 104.89 (7.53) | 104.59 (8.22) | 104.37 (6.19) | 102.26 (7.44) | 0.472 |
| Glucose, mg/dL | 140.00 [106.00, 180.25] | 149.50 [119.50, 174.25] | 151.00 [110.50, 179.00] | 133.00 [105.50, 160.00] | 0.574 |
| Bicarbonate, mmol/L | 24.56 (6.52) | 24.40 (6.61) | 24.26 (6.62) | 24.78 (8.11) | 0.994 |
| **Charlson comorbidity** |  |  |  |  |  |
| Myocardial infarct, n (%) |  |  |  |  | 0.700 |
| No | 75 (93.75) | 74 (94.87) | 19 (100.00) | 25 (92.59) |  |
| Yes | 5 (6.25) | 4 (5.13) | 0 (0.00) | 2 (7.41) |  |
| Peripheral vascular disease, n (%) |  |  |  |  | 0.608 |
| No | 80 (100.00) | 77 (98.72) | 19 (100.00) | 27 (100.00) |  |
| Yes | 0 (0.00) | 1 (1.28) | 0 (0.00) | 0 (0.00) |  |
| Cerebrovascular disease, n (%) |  |  |  |  | 0.004 |
| No | 78 (97.50) | 77 (98.72) | 15 (78.95) | 25 (92.59) |  |
| Yes | 2 (2.50) | 1 (1.28) | 4 (21.05) | 2 (7.41) |  |
| Dementia, n (%) |  |  |  |  | 0.688 |
| No | 77 (96.25) | 76 (97.44) | 18 (94.74) | 27 (100.00) |  |
| Yes | 3 (3.75) | 2 (2.56) | 1 (5.26) | 0 (0.00) |  |
| Chronic pulmonary disease, n (%) |  |  |  |  | 0.370 |
| No | 63 (78.75) | 60 (76.92) | 18 (94.74) | 22 (81.48) |  |
| Yes | 17 (21.25) | 18 (23.08) | 1 (5.26) | 5 (18.52) |  |
| Rheumatic disease, n (%) |  |  |  |  | > 0.999 |
| No | 79 (98.75) | 77 (98.72) | 19 (100.00) | 27 (100.00) |  |
| Yes | 1 (1.25) | 1 (1.28) | 0 (0.00) | 0 (0.00) |  |
| Peptic ulcer disease, n (%) |  |  |  |  | 0.608 |
| No | 80 (100.00) | 77 (98.72) | 19 (100.00) | 27 (100.00) |  |
| Yes | 0 (0.00) | 1 (1.28) | 0 (0.00) | 0 (0.00) |  |
| Mild liver disease, n (%) |  |  |  |  | 0.199 |
| No | 74 (92.50) | 77 (98.72) | 19 (100.00) | 26 (96.30) |  |
| Yes | 6 (7.50) | 1 (1.28) | 0 (0.00) | 1 (3.70) |  |
| Severe liver disease, n (%) |  |  |  |  | > 0.999 |
| No | 79 (98.75) | 77 (98.72) | 19 (100.00) | 27 (100.00) |  |
| Yes | 1 (1.25) | 1 (1.28) | 0 (0.00) | 0 (0.00) |  |
| Diabetes, n (%) |  |  |  |  | 0.518 |
| No | 71 (88.75) | 66 (84.62) | 18 (94.74) | 25 (92.59) |  |
| Yes | 9 (11.25) | 12 (15.38) | 1 (5.26) | 2 (7.41) |  |
| Paraplegia, n (%) |  |  |  |  | — |
| No | 80 (100.00) | 78 (100.00) | 19 (100.00) | 27 (100.00) |  |
| Yes | — | — | — | — |  |
| Renal disease, n (%) |  |  |  |  | 0.949 |
| No | 68 (85.00) | 68 (87.18) | 17 (89.47) | 23 (85.19) |  |
| Yes | 12 (15.00) | 10 (12.82) | 2 (10.53) | 4 (14.81) |  |
| Malignant cancer, n (%) |  |  |  |  | 0.046 |
| No | 69 (86.25) | 73 (93.59) | 19 (100.00) | 27 (100.00) |  |
| Yes | 11 (13.75) | 5 (6.41) | 0 (0.00) | 0 (0.00) |  |
| Metastatic solid tumor, n (%) |  |  |  |  | 0.608 |
| No | 80 (100.00) | 77 (98.72) | 19 (100.00) | 27 (100.00) |  |
| Yes | 0 (0.00) | 1 (1.28) | 0 (0.00) | 0 (0.00) |  |
| Aids, n (%) |  |  |  |  | 0.226 |
| No | 80 (100.00) | 78 (100.00) | 19 (100.00) | 26 (96.30) |  |
| Yes | 0 (0.00) | 0 (0.00) | 0 (0.00) | 1 (3.70) |  |
| **Vital signs** |  |  |  |  |  |
| RR, /min | 29.23 (8.74) | 24.36 (7.74) | 23.74 (9.00) | 18.85 (5.38) | < 0.001 |
| HR, /min | 104.20 (22.96) | 95.63 (22.23) | 95.16 (25.69) | 82.37 (16.33) | < 0.001 |
| ROX | 4.11 [2.99, 5.50] | 4.85 [3.50, 6.36] | 4.18 [3.13, 4.66] | 11.90 [9.79, 14.98] | < 0.001 |
| **Mortality outcome** |  |  |  |  |  |
| 28-day mortality, n (%) |  |  |  |  | 0.137 |
| No | 52 (65.00) | 58 (74.36) | 17 (89.47) | 21 (77.78) |  |
| Yes | 28 (35.00) | 20 (25.64) | 2 (10.53) | 6 (22.22) |  |

Data are presented using mean (standard deviation (SD)), median (lower quartile-upper quartile), or number (proportion, %). ICU: intensive care unit; SOFA: Sequential Organ Failure Assessment; APS-III: Acute Physiology Score III; GCS: Glasgow Coma Scale; WBCs: white blood cells. INR: international normalized ratio; PTT: partial thromboplastin time; BUN: blood urea nitrogen; RR: respiratory rate; HR: heart rate; ROX: respiratory rate-oxygenation.
